# The Influence of the Composition of a Water–Alcohol Solution on the Synthesis of Nanostructures Using a Steam-Water Electric Arc Plasma Torch

**DOI:** 10.3390/nano16070409

**Published:** 2026-03-28

**Authors:** Antonina I. Karlina, Andrey E. Balanovskiy, Georgy E. Kurdyumov, Vitaliy A. Gladkikh, Galina Yu. Vitkina, Roman V. Kononenko, Viktor V. Kondratiev, Yulia I. Karlina

**Affiliations:** 1 Scientific Research and Testing Center “Stroytest”, Moscow State University of Civil Engineering, Moscow 129337, Russiajul.karlina@gmail.com (Y.I.K.); 2 Department of Materials Science, Welding and Additive Technologies, Irkutsk National Research Technical University, Irkutsk 664074, Russia; 3 Advanced Engineering School, Cherepovets State University, Lunacharsky Street, 5, Cherepovets 126600, Russia; 4 Institute of Information Technology and Data Science, Irkutsk National Research Technical University, 83 Lermontov St., Irkutsk 664074, Russia; 5 Innovation and Technology Center for Energy and Resource Conservation, A. P. Vinogradov Institute of Geochemistry of the Siberian Branch of the Russian Academy of Sciences, Irkutsk 664033, Russia

**Keywords:** fullerenes, graphene, nanotubes, alcohol plasma torch, voltage, current, gas pressure, current source, cathode deposit, dispersion, evaporation, fragmentation

## Abstract

Nanostructured products synthesized using electric arc vapor plasma with various alcohol solutions exhibiting very high enthalpy and low mass flow rates in a direct current discharge in direct contact with a vapor vortex surrounding the arc column were studied. The nanostructured products obtained in our experiments with various alcohol solutions (ethanol, propanol, and benzene) were analyzed using modern nanostructure identification methods. The diameters of the synthesized multi-walled carbon nanotubes (MWNTs) ranged from 9 to 35 nm, single-walled carbon nanotubes (SWNTs) from 2 to 4 nm, and graphene flakes from 1 to 7 sheets, depending on the alcohol solution composition. Fullerene-like structures identified by HRTEM were obtained from a benzene mixture using electric arc vapor plasma synthesis. It is shown that the thermal steam plasma process with various alcohol solutions has great potential for the synthesis of nanotubes and graphene flakes due to the continuous and easy-to-implement method, cheap raw materials and adjustable carbon content due to the combination of different mixture compositions.

## 1. Introduction

One of the pressing problems of modern materials science is the synthesis and development of new materials with specified physicochemical properties. Among such materials, particular attention is attracted by composite materials based on the use of polymers and carbon nanostructures, a special place among which is occupied by carbon nanotubes (CNTs), nanofibers, nanorods [1,2,3], fullerenes [4], graphene [5], etc. [6,7,8]. Carbon nanotubes have been the subject of long-term scientific interest of a large number of researchers since their official discovery in 1992 [9,10]. Although they were discovered unofficially [11], the results were published in the open press in 1952 [12,13]. The historical chronology of the discovery of CNTs can be found in the works [11,14,15]. There are several definitions of CNTs. We present the definition of CNTs given by E.G. Rakov [16]: “nanotubes are thread-like nanoparticles of carbon atoms or other elements containing an extended internal cavity.” CNTs are seamless cylindrical structures formed by rolled graphene sheets and can be arranged as single-walled (SWCNTs) or multi-walled (MWCNTs) nanotubes. SWCNTs typically range from 0.4 to 2 nm in diameter. MWCNT outer and inner diameters range from 2 to 100 nm and from 1 to 20 nm [16,17,18,19,20,21,22]. Over three decades, a large number of CNT synthesis methods have been proposed. CNT synthesis methods can be classified according to carbon atomization methods into physical and chemical, based on the catalytic decomposition of carbon-containing compounds. Several basic methods are currently used for the synthesis of CNTs, the main ones being electric arc [3,4,9,10], laser ablation [17,18], and catalytic pyrolysis of hydrocarbons [16,19,20,21]. The most common method for producing carbon nanotubes is synthesis in arc discharge plasma between graphite electrodes in a helium atmosphere.

The essence of the electric arc method or the arc discharge method is the sputtering of a graphite anode in an inert atmosphere and the deposition of carbon material on the cathode, as in the synthesis of fullerenes [1,2,3,4]. However, the conditions of the synthesis process differ in that the synthesis of (CNTs) occurs at low arc discharge current densities and high pressure of the inert atmosphere with a larger cathode diameter than in the synthesis of fullerenes [3,4,10]. The presence of iron group metals in the graphite anode has a catalytic effect on the formation of carbon nanotubes and contributes to an increase in the yield of (CNTs) up to 60% [9,10,11]. The carbon deposit formed on the cathode and the chamber walls, along with carbon nanotubes, contains various forms of carbon particles (carbon soot). The factors influencing the process stability and the quality characteristics of CNTs are voltage, current density, plasma temperature, total system pressure, inert gas feed rate, reaction chamber dimensions, synthesis duration, presence and geometry of cooling devices, nature and purity of electrode material, ratio of their geometric dimensions, as well as a number of parameters that are difficult to quantify, such as the rate of carbon vapor cooling, etc. A distinctive feature of the synthesis method under consideration (CNTs) is that it allows one to obtain the highest quality nanotubes up to several micrometers long with similar morphological properties and a diameter of 1 to 5 nm [16]. However, it should be noted that achieving such high quality is associated with significant technological difficulties, primarily related to the need for multi-stage purification of the product from soot inclusions and other impurities [16]. The main disadvantage of this method is the difficulty of creating a continuous process. In addition, the process is accompanied by the formation of a large number of impurities of amorphous carbon, fullerenes, and graphitized particles, which leads to a low yield of target (CNTs) (in the case of single-walled carbon nanotubes, the yield does not exceed 20–40%) and requires their multi-stage purification.

The laser ablation method [17,18] is based on the evaporation of a graphite-containing target in a high-temperature reactor. During synthesis, a laser beam is focused on a target containing metal and graphite in a heated inert gas atmosphere. The carbon particles formed during the synthesis are deposited on the cooled surface of the reactor and on a collector. This method primarily synthesizes single-wall carbon nanotubes. Compared to the arc discharge method, direct evaporation allows for more precise control of growth conditions and the production of high-quality nanotubes. Furthermore, the CNT yield is approximately 70%.

Chemical vapor deposition (CVD) [16,18,19,20,21] is based on the pyrolysis of carbon-containing gases, vapors, liquids, and solids (e.g., polymers). Carbon nanotubes are deposited on a substrate with metal catalyst particles, and the size of these metal particles determines the nanotube diameter. The growth mechanism of CNTs in this method involves the thermal decomposition of a carbon-containing precursor and the dissolution of the resulting carbon in a metal nanoparticle. As the carbon concentration in the metal catalyst particle increases, conditions are created under which the release of excess carbon into a hemifullerene, which is the end of the CNT, becomes energetically more favorable. The excess carbon is then consumed to form carbon–carbon bonds, and the hemisphere rises from the melt, transforming into a cylindrical structure. Modern synthesis methods make it possible to obtain materials with a high CNT content of up to 90–95% by weight [16]. It should be noted that carbon nanotubes synthesized by CVD differ significantly from those produced by arc and ablation methods. They typically contain a greater number of defects, have a wide range of diameters and lengths, and exhibit larger interlayer distances. Therefore, despite the apparent simplicity of the technology, pyrolytic synthesis methods require a careful approach to selecting the parameters used and studying and optimizing the kinetic characteristics of the process [16].

A review of the literature shows that most research is focused on developing a simple and inexpensive method for producing nanomaterials. An electrolytic method was proposed in [22] by passing a current of 3–5 A between two graphite electrodes (a graphite crucible served as the anode and a graphite rod as the cathode). A chemical method for producing nanotubes is described in [23]. An improvement on the non-catalytic (although silicon is present) pyrolytic method, which involves heating silicon nitride nanograins in a boron nitride crucible to 1200–1900 °C in nitrogen in a graphite furnace, is presented in [24].

A flame synthesis method for producing single-walled nanotubes using a simple laboratory diffusion flame was demonstrated in [25]. In this method, a hydrocarbon mixture is fed in a stream onto a tantalum filament heated to 2200 °C. The entire chamber is maintained under a dynamic vacuum of 40 Torr. According to the authors, this should result in the formation of single-walled carbon nanotubes.

A continuous synthesis of highly crystalline carbon nanotubes was proposed in [26]. This was achieved by varying the injection zone configuration in a reactor used in floating-catalyst chemical vapor deposition (FC-CVD). The production rate was 1.2 mg/min, and the carbon content in the deposit reached 70%.

A new method for the synthesis and spinning of carbon nanotube fibers using floating-catalyst chemical vapor deposition (FC-CVD) in an open atmosphere without hydrogen as a carrier gas was presented in [27]. In recent years, a thermal plasma process at atmospheric pressure has been developed for the continuous synthesis of graphene flakes [28,29,30]. A carbon-containing precursor is fed directly into the thermal plasma region, where the precursor decomposes into smaller reactive fragments. These reactive fragments then recombine in the plasma environment, forming graphene flakes and other products. In [28,29], a methane decomposition method using a magnetically rotating arc plasma system was proposed for the synthesis of graphene flakes. Carbon nanomaterials are synthesized in various buffer gases (Ar, He, Ar-H_2_, and Ar-N_2_) by propane decomposition.

The paper [29] presents a review of plasma methods for producing nanostructured particles. Plasma synthesis of graphene using various sources such as radio frequency (RF) plasma, direct current (DC) plasma, and microwave (MW) plasma is a promising method due to its controllability, flexibility, and scalability. Among the above-mentioned plasma sources, microwave plasma-based synthesis is rapidly gaining popularity. This method, applicable both in vacuum and at atmospheric pressure, offers many advantages over other methods, which further increases its attractiveness in the field of graphene production. It should be noted that, currently, the production of nanomaterials by flame aerosol synthesis (gas, plasma) can be divided into two main categories depending on the precursor supply conditions: vapor-assisted flame aerosol synthesis (VAFS) and liquid-assisted flame aerosol synthesis (LAFS) (using a nebulizer or atomizer). Most studies [31,32,33,34,35,36,37,38,39,40,41,42] classify this process as gas combustion synthesis, as nanoparticles are formed directly from the gas phase through a self-sustaining reaction of gaseous substances. However, many authors [31,32,33,34,35,36,37,38,39] note that producing high-quality graphene in a continuous, closed cycle with high productivity remains a complex task. Production economics must also be considered. In [43,44], a hybrid method of arc discharge and laser ablation is considered with an emphasis on their features in the production of carbon nanotubes. The final product in the form of a cathode deposit in the arc discharge method consists of carbon nanotubes and various carbon impurities. Analysis of carbon nanotubes obtained in commercially available arc discharge systems shows that commercial cathode deposits contain about 10% nanotubes, where the majority of carbon nanotubes are multi-walled carbon nanotubes [45]. Considering that the quality of the final product depends on the parameters of the carbon-plasma jet, it is possible to increase the synthesis yield by controlling the plasma jet. (MWCNTs). At the same time, it should be noted that these two synthesis methods are not continuous and require a period of operation stoppage for reloading new graphite electrodes (arc discharge) or new graphite rods (laser ablation) and for collecting carbon-containing products inside the devices

In the work [46], multi-walled carbon nanotubes were synthesized using a hybrid method of arc discharge and chemical vapor deposition. Multi-walled carbon nanotubes were synthesized on a thin nickel film deposited by magnetron sputtering on a silicon substrate, and then thermal chemical vapor deposition using acetylene at a temperature of 750 °C. The current in the arc discharge method varied in the range of 50–200 A. No catalyst was used in the carbon nanotube synthesis process, so purification of the grown CNTs was not required. Chemical vapor deposition is considered the most promising process for obtaining nanotubes integrated into a device directly during the synthesis process [47,48,49].

It is worth noting that methane is widely used as a carbon source for the production of carbon nanomaterials, other common precursors of CNTs include ethylene, acetylene, benzene, xylene, and carbon monoxide. The use of gaseous hydrocarbons instead of a consumable graphite electrode makes this method continuous and scalable for industrial production of carbon black and nanotubes [50,51,52]. A variation of the CVD method is the use of plasma as a means of delivering energy to the carbon source to drive dissociation reactions, known as plasma-enhanced chemical vapor deposition (PECVD), which enables the nucleation and growth of carbon nanotubes in floating or stabilized catalysts inside a plasma reactor chamber.

In [53,54], the thermophysical characteristics of a plasma-water arc jet are considered; water (100%) or water (50%) + ethanol (50%) were used as the plasma-forming medium (in the form of steam). The authors note the promise of water-steam plasma torches for obtaining nanomaterials. Some studies have noted the positive effect of water on the growth of nanotubes [55,56,57]. The supply of H_2_O vapor with a concentration of 200 ppm at 650 °C accelerated the initial growth rate and increased the average lifetime of catalytically active centers. According to the authors [57], water vapor is present in the system as a mild oxidizer, the purpose of which is to remove amorphous carbon formed during the synthesis process from the synthesis chamber without damaging the CNTs. The quality and height of the resulting CNTs can be significantly improved by adding an appropriate amount of water vapor, but it is important to determine the optimal amount.

Among the various types of thermal plasma, steam thermal plasma is characterized by high enthalpy and high redox capacity, and is also environmentally friendly. A key advantage of steam flame synthesis is the production of high-purity products with well-controlled crystallinity, ultrafine diameters, and narrow size distributions.

According to [58], soot, being the main combustion product in the condensed phase, is formed as a result of incomplete combustion of hydrocarbon fuels. In most cases, the presence of soot indicates poor combustion conditions, under which not all fuel molecules can be fully oxidized to form CO_2_ and H_2_O for maximum thermal energy extraction. Soot particles contain chemical energy in the form of CH and CC bonds, which could be released as the desired thermal energy if soot formation were completely avoided or if all initially formed soot particles were completely oxidized at a later stage. The authors [58,59] believe that flame synthesis is based on the incomplete combustion of carbon-containing raw materials in the presence of a catalyst. Flame synthesis, like traditional methods, requires a carbon source, a catalyst, and heat, with the heat supply in this case provided directly by the combustion reaction.

A literature review revealed that there are virtually no published works on the synthesis of CNTs, graphene, and other nanomaterials using electric arc vapor plasma to convert carbon and produce CNTs, graphene, and other nanomaterials. In excellent papers and reviews of plasma synthesis methods [30,31,32,33,34,35,36,37,60,61,62], high-purity carbon nanotubes are synthesized by decomposing methane using high-temperature arc plasma (5000–20,000 K). At the same time, we found only one work where water plasma with controlled fog formation obtained using four different solutions, water, 1-propanol, 2-propanol, and ethanol solutions [63], was used to study the arc characteristics. The work presented an experimental setup for research, where fog (from solutions of propanol, ethanol, and water) was created using ultrasound.

However, we were surprised by the fact that the authors [63] did not indicate that soot should form on the cathode. This well-known case is well described in the operating instructions for water-steam plasma torches such as the Multiplaz 3500 and Pzlaar for brazing. The manufacturers of these plasma torches recommend operating on water (100%) or on a mixture of water (50%) and ethanol (50%). If the ethanol content in the mixture is greater than 50%, soot will form on the cathode. The works [64,65] consider the operation of a plasma torch with plasma consisting of 100% water vapor. A source of atmospheric pressure microwave plasma operating on water vapor is proposed in the work [66] and has many potential applications.

This study was the first to experimentally investigate the types of solid deposits formed using various alcohol solutions using a Multiplaz 3500 industrial water plasma torch with controlled carbon-containing vapor generation. The scientific innovation lies in the use of plasma consisting of alcohol vapor. Alcohol vapor enters the arc gap due to intense evaporation during its heating in the expansion tank and due to natural pressure.

The resulting solid cathode deposit samples were characterized using scanning electron microscopy (SEM), transmission electron microscopy (TEM), X-ray diffraction (XRD), and other methods.

## 2. Materials and Methods

### 2.1. Experimental Setup

**Figure 1:**
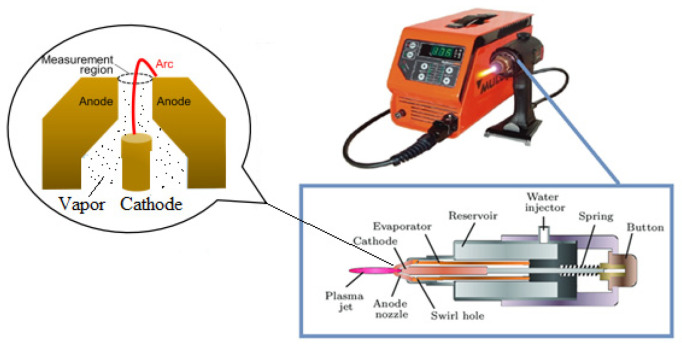
Configuration of the steam plasma torch and equipment for CNTs synthesis.

The steam-water plasma system with controlled vapor generation is shown in Figure 1. The portable industrial plasma unit “Multiplaz-3500” (Shenzhen, China) is designed for manual plasma cutting, welding, brazing, and soldering (brazing of dissimilar materials) of ferrous and non-ferrous metals, including alloyed and unalloyed steels, stainless steels, cast iron, copper group metals, aluminum, and its alloys. The portable plasma unit “Multiplaz-3500” generates low-temperature plasma obtained by heating the working fluid (water (100%)) or a water–alcohol mixture (water (80%) + alcohol (20%)) to the ionization temperature. The mixture is poured into the torch before the welding operation (Figure 1). The unit is designed for continuous operation (duty cycle coefficient PV = 100%).

The device consists of a plasma torch and a power supply. Plasma torch (Figure 1). The plasma torch is a coaxial DC torch with a non-transferable arc discharge, a hafnium cathode embedded in a copper rod (outer diameter: 8 mm, height: 20 mm), and a nozzle-type copper anode (nozzle outlet diameter: 2 mm). Hafnium with a diameter of 1.0 mm is pressed into the cathode material and prevents erosion and promotes increased operating time in oxidizing conditions. Operating principle of the plasma torch. After filling the torch with working fluid through a special hole (Figure 1), the power supply is turned on and voltage is applied to the cathode to excite the arc. This causes a short circuit between the cathode and the torch nozzle. When the start button is released, an electric arc is generated between the cathode and the nozzle. The arc energy heats the nozzle, which heats the evaporator, which heats the working fluid, converting it into steam. The resulting steam passes through a vortex orifice in the evaporator and enters the arc chamber as a vortex gas. As the amount of steam increases, a steam plasma jet forms at the nozzle outlet.

The torch thus operates in a highly efficient mode, as the water cools the electrode and simultaneously acts as the plasma-forming gas. Liquid flow is approximately 0.25 L/h at an arc power of 1 kW. The torch weighs 0.9 kg and is powered by IGBT DC power sources with a maximum output voltage of 220 ÷ 250 V. The output current (3 ÷ 9.5 A) can be adjusted using the current regulator on the device. Steam, under internal pressure (0.4 ÷ 1.2 atm), rushes toward the outlet in the nozzle. As it exits the nozzle, the steam compresses the electric arc. This compression of the electric arc increases its temperature. The compressed electric arc heats the steam to the ionization temperature. The device operates in two modes. The electric arc burns between the cathode and the nozzle. The plasma jet is the only energy transfer agent to the workpiece. To study the effect of electric arc steam on cathode deposit formation, alcohol solutions were used: 1-propanol, 2-propanol, ethanol, and benzene. Solution concentrations were adjusted throughout the experiment to 5 mol.% (purity: 99.8%). Guaranteed reagent (LLC “Sintezspirt”, Orsk, Russia).

### 2.2. Research Methods

A Tektronix TDC 1012B oscilloscope (Beaverton, OR, USA) was used to measure current and voltage. The pressure in the plasma torch nozzle was measured using the technique described in [63,64,65]. Carbon nanotube samples were studied using a DRON-7 diffractometer (Saint-Petersurg, Russia) in conventional Bragg–Brentano geometry (*θ*–2*θ*) with CuKα radiation (with a beta filter, tube mode 40 kV/20 mA). In the Bragg–Brentano geometry, a diffraction pattern was first recorded in the 5–165° range (overview), followed by a diffraction pattern in the 5–100° range with a step of 0.02° and an exposure time of 5 s, scanned four times. The data from the four measurements were summed (averaged). Spectra were processed using MAUD (Materials Analysis Using Diffraction) v.2.33. An open source database was used as the crystallographic structure database. The structural properties of CNTs were studied using Raman spectroscopy on a DFS-52 spectrometer (Tokyo, Japan) in photon-counting mode with a scan step of 1 cm^−1^ at room temperature in reflection geometry. The radiation source was a 532 nm laser with a power of 150 mW. The surface state of carbon nanotube samples was studied using IR spectroscopy on a Vector-22 infrared (IR) Fourier transform spectrometer (Bruker, Ettlingen, Germany), with an optical resolution of 4 cm^−1^ and accumulation of 32 scans in the range of 4000–400 cm^−1^.

The JIB 4500 multi-beam scanning electron microscope (Tokyo, Japan) for sample processing (preparation) and observation features both a scanning electron microscope (SEM) with a LaB6 electron gun (Tokyo, Japan) for high resolution and long life, and a focused ion beam (FIB) unit for high resolution and high processing speed. The Tecnai™ G^2^ F20 transmission electron microscope (Tokyo, Japan) offers excellent imaging performance in TEM, STEM, and nanoanalysis modes, ultra-high vacuum, high spatial coherence, a full sample preparation range, an information limit of <0.12 nm, and a magnification of 25×–1000 kHz (TEM) and 150×–230 MHz (STEM). The instrument’s performance is further enhanced by the addition of EDS (energy dispersive X-ray spectrometer) analyzers (Tokyo, Japan).

## 3. Results and Discussion

**Figure 2:**
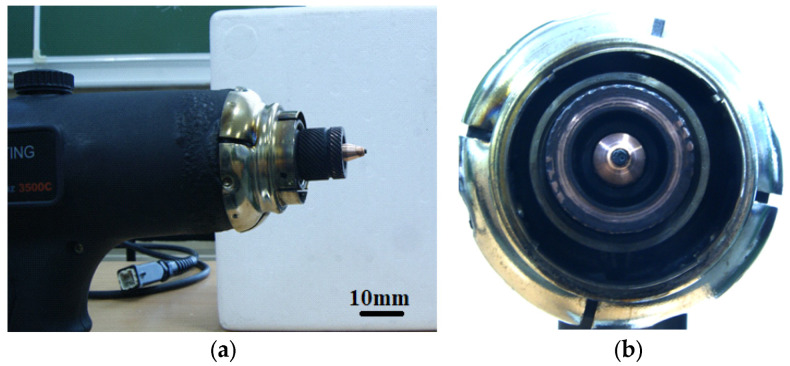
Photograph of the cathode deposit on the cathode surface after operation in a mixture (30% water + 70% ethanol). (**a**) side view of the cathode deposit; (**b**) front view.

**Figure 3:**
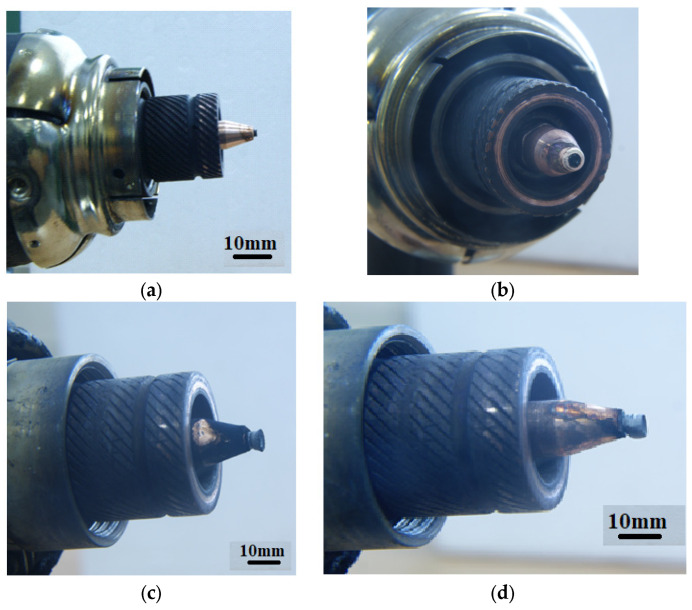
Photograph of the cathode deposit on the cathode surface after operation in a mixture of (20% water + 80% ethanol) (**a**,**b**), and (**c**) (10% + 90% ethanol), (**d**) (100% ethanol).

**Figure 4:**
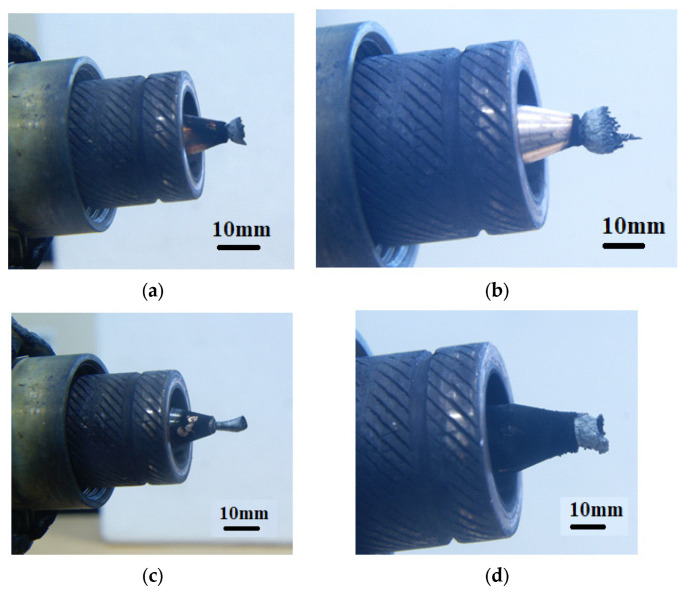
Photograph of the cathode deposit on the cathode surface after operation in a mixture of (**a**) (30% water + 70% propanol), (**b**) (10% water + 90% propanol), (**c**) (100% propanol), (**d**) 100% benzene.

Experimental studies of the reaction products confirmed that a dense deposit material does indeed form on the cathode (Figure 2). The deposit is clearly formed as a cone-shaped cylinder. As the ethanol content in the mixture increases, the size of the cathode deposit increases (Figure 3). Switching to a different alcohol (propanol) shows an increase in the cathode deposit (Figure 4a,b). Using 100% benzene to produce the vapor mixture showed the highest amount of carbon on the cathode surface (Figure 4d).

**Figure 5:**
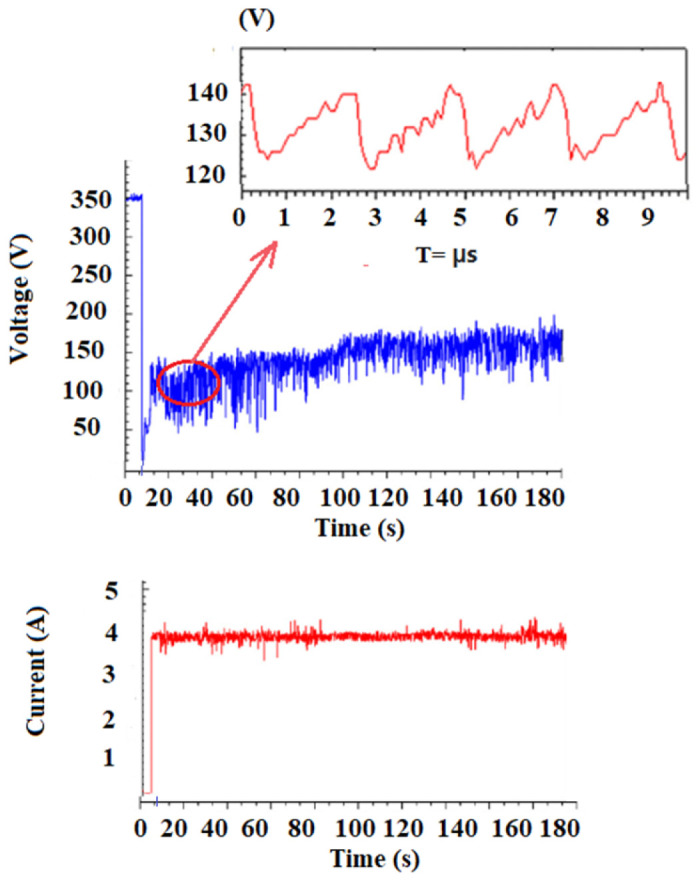
Current and voltage values in a steam arc (20% water, 80% ethanol).

**Figure 6:**
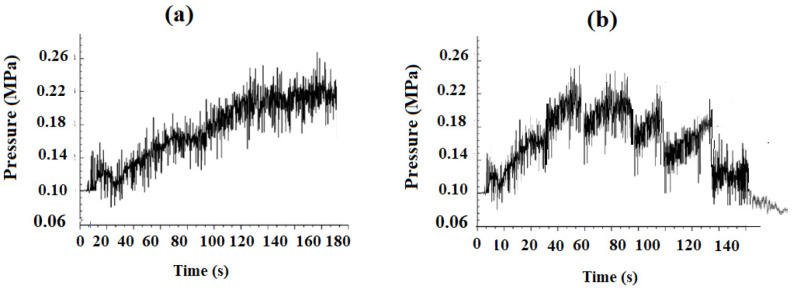
Pressure values in the plasma torch nozzle (**a**) 100% water and (**b**) (20% water, 80% ethanol).

**Figure 7:**
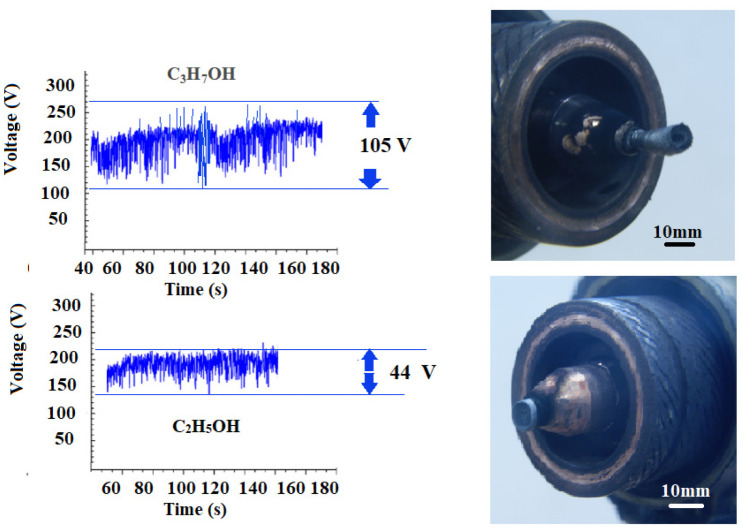
Voltage fluctuations in steam plasma for different alcohols (ethanol, propanol) and the appearance of the deposit formed on the cathode.

The results of measuring the electrical characteristics of the vapor discharge are presented in Figure 5, Figure 6 and Figure 7. Voltage fluctuations (sawtooth type, Figure 5) in a plasma torch with a self-setting electric arc length are well known for DC plasma torches with cylindrical and conical channels of plasma torch nozzles [67,68,69]. It is known that the electric arc in a plasma torch [67,68,69], starting at the cathode located on the channel axis, can spontaneously close at any point on the anode. The flow movement and the transverse blowing of the working gas over the section of the arc closing on the wall lead to stretching, breaking and reconnection of the arc to a new location on the anode. The reconnection phenomenon generates arc voltage fluctuations; it promotes more efficient gas heating and increases the efficiency of the plasma torch. It has been experimentally established [67,68,69] that the plasma arc combustion process is accompanied by voltage fluctuations of various natures. Firstly, there are spontaneous voltage fluctuations caused by gas ionization processes. The amplitude of these fluctuations is determined primarily by the type of plasma-forming gas. Secondly, there are voltage fluctuations caused by external disturbances—changes in the arc gap length, plasma-forming gas flow rate, etc. [68]. Explanations of the physical mechanisms leading to the occurrence of arc voltage fluctuations in such plasma torches [67,68,69] are insufficient and do not allow us to obtain quantitative dependences of the observed fluctuations on the main parameters of the arc discharge.

With regard to our experiments, a detailed analysis of the nature of combustion voltage fluctuations and the shunting mechanism was given in [63]. In [68,69], it was shown that the nature of shunting is electrodynamic in nature (although gas-dynamic processes also play an important role, Figure 6b), and the amplitude of voltage fluctuations and frequency are associated with the processes of elongation of the current cord and shunting of the electric arc onto the anode wall. Gas-dynamic processes influence the shunting mechanism of the electric arc. Bending of the section of the current cord, initially in position and adjacent to the anode spot, changes the nature of the interaction of different sections of the current cord. The length of the arc from the nozzle outlet in all cases clearly increased with increasing arc voltage, which is a result of the resistance force pushing the arc downstream under the action of high pressure inside the torch. It is known that the average arc voltage in alcohol solutions can be mainly dependent on the vapor pressure, since it is caused not only by similar values of surface tension and viscosity, but also by the volatile properties [63,64]. In [68], it was found that the vapor pressure depends exponentially on the temperature of the aqueous mixture. According to the explanation in [63,64], the vapor pressure mainly depends on intermolecular forces. In alcohol solutions, there are two types of intermolecular forces: hydrogen bonds in the form of dipole–dipole attraction of hydroxyl groups and dispersion forces, where the dispersion forces are related to the molecular structure [64]. Hydrogen bonds are the main intermolecular force in both alcohol solutions; however, ethanol has a shorter chain than propanol, which makes its dispersion force weaker. Therefore, ethanol has a higher vapor pressure, which leads to a higher rate of vapor formation and an increase in the average arc voltage. At the same time, we see that the amplitude of voltage fluctuations is higher for propanol than for ethanol (Figure 7). The cathode deposit is also evidently larger. The vapor pressure of propanol was higher than that of ethanol. As a first approximation, we can conclude that higher vapor pressure at higher solution temperatures promotes the formation of more vapor inside the torch between alcohol solutions compared to water, resulting in a longer arc. Ultimately, this leads to a higher average arc voltage, corresponding to a greater resistance force.

**Figure 8:**
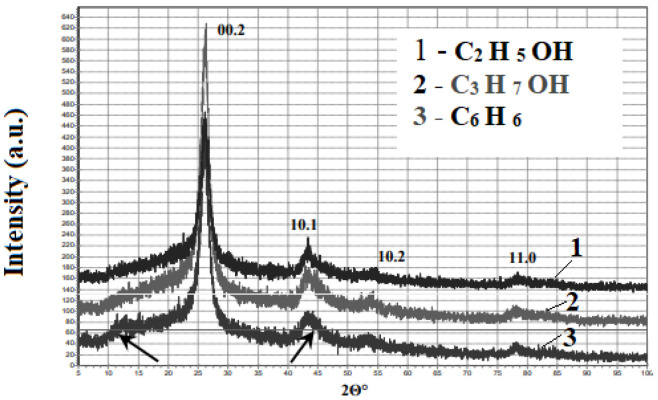
X-ray diffraction patterns of cathode deposits.

X-ray diffraction patterns of cathode deposits were made (Figure 8). Sample synthesis modes. Current 4A, voltage 165 V. The peak in the diffraction pattern of sample 3 at 2*θ*~12° indicates the presence of fullerene in it [1,2,3,4,5,6,7,8,9,10], and the symmetry of the diffraction reflection 10.1 for sample 1 indicates its homogeneity [16,17,18,19], in contrast to samples 2 and 3, which are characterized by the asymmetry of this reflection.

From Figure 8, it is evident that all the diffraction patterns are characterized by broad maxima at small angles (less than 26°), a narrow peak at 2*θ*~26°, usually associated with graphite-like carbon, and a set of reflections in the region of 45° and above. High-angle (45° degrees and above) reflections are predominantly of impurity origin. As is known [16,17,18,19,20,21,22], the diffraction pattern of single-wall nanotubes can contain information about the perfection of both the nanotubes themselves and the bundles they form. When representing single-wall nanotubes as close-packed cylinders forming a two-dimensional triangular lattice [10,11,12,13,14,15,16,17,18,19,20], which determines the small-angle scattering in the diffraction pattern in the initial angle range of 5–20°, the most intense peak C (10) is expected at 5°, which corresponds to the initial decline in the obtained diffraction patterns. The peak at 2*θ*~8.6° is attributed to the reflection C (11), corresponding to an interplanar distance of 10.27 Å. The reflection at 2*θ*~26.65° corresponds to (002) graphite, but is also usually attributed to the close-packed carbon structure formed in the SWCNT bundles.

**Figure 9:**
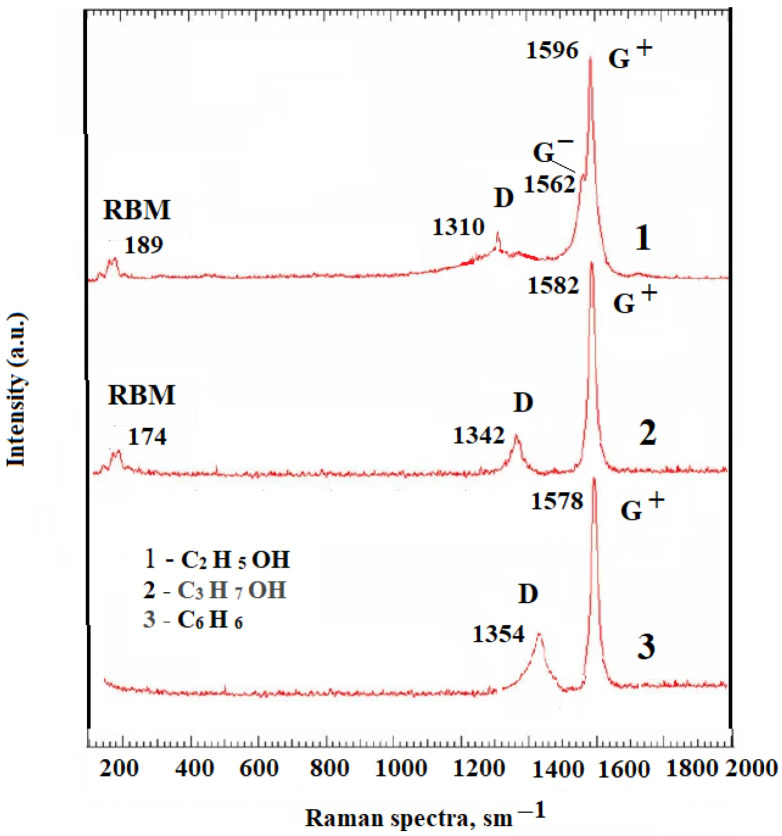
Raman spectroscopy of cathode deposit (ethanol 100%).

**Figure 10:**
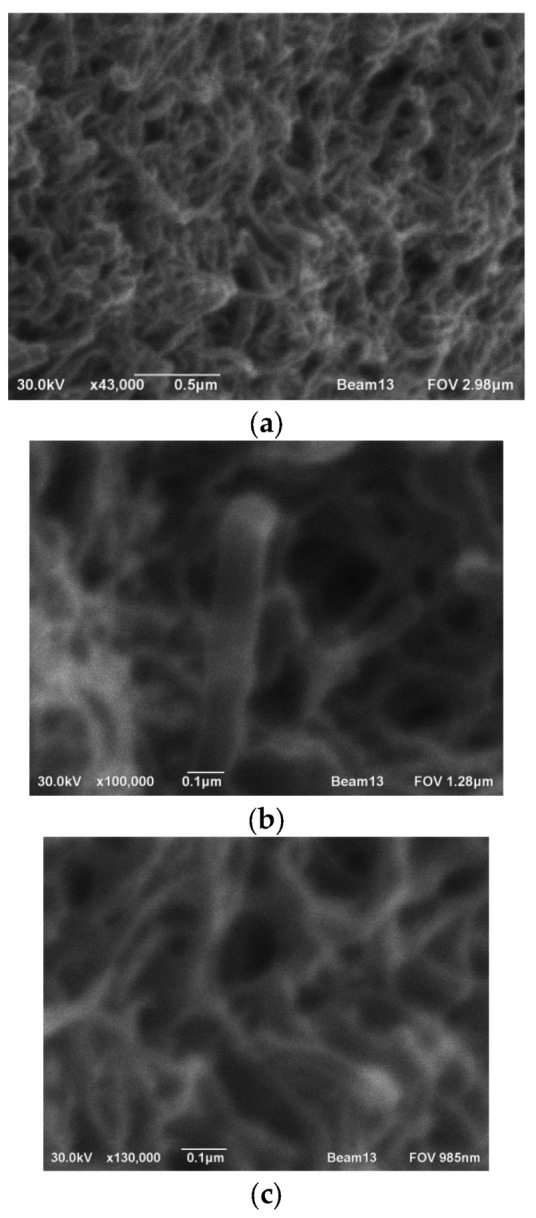
Electron photographs of the cathode deposit (ethanol 100%) at different magnifications. (**a**)—loose cathode deposit, nanotubes visible; (**b**,**c**)—dense part of the cathode deposit at a magnification of over 100,000×.

Raman spectroscopy [8,9,10,11,12,13,14,15,16,17,18,19,20,21,22,23,24,25] is widely used to study carbon nanostructures and provides information on the structural features of nanotubes (the presence of defects, impurities, nanotube diameter, and chirality). When carbon nanotubes are irradiated with light, photons are absorbed, causing phonon vibrations of the carbon atoms in the lattice, directed along and perpendicular to the axis. These vibrations are recorded in the Raman spectra as the G-band (tangential mode) (1578–1592 cm^−1^), which determines 53 vibrations of carbon atoms (sp2) and reflects the degree of crystallization. Due to the localization and curvature of the graphene layers of CNTs, the G-band acquires an asymmetric appearance in multi-walled carbon nanotubes and splits into two bands, G+ and G−, for single-walled ones. Figure 9 shows the Raman spectrum of the original sample obtained during the formation of a cathode deposit from steam (ethanol 100%). As can be seen from the figure, there are two peaks corresponding to the disordered (D-band in the region of 1320–1350 cm^−1^) and ordered phases (G-band in the region of 1540–1590 cm^−1^). The morphology of the synthesized cathode deposits with different vapor plasma compositions was characterized using SEM, which demonstrated the successful formation of nanostructured objects. Figure 10 shows characteristic nanotube clusters at various magnifications. Morphological analysis of the sample with pure benzene revealed a higher number of graphene layers compared to the ethanol and propanol sample. Electron microscopic photographs of cathode deposits using other types of alcohol are presented in the Supplementary Materials to the article.

More detailed images of the cathode deposit nanostructures are presented below, where the morphology is examined using high-resolution transmission microscopy.

**Figure 11:**
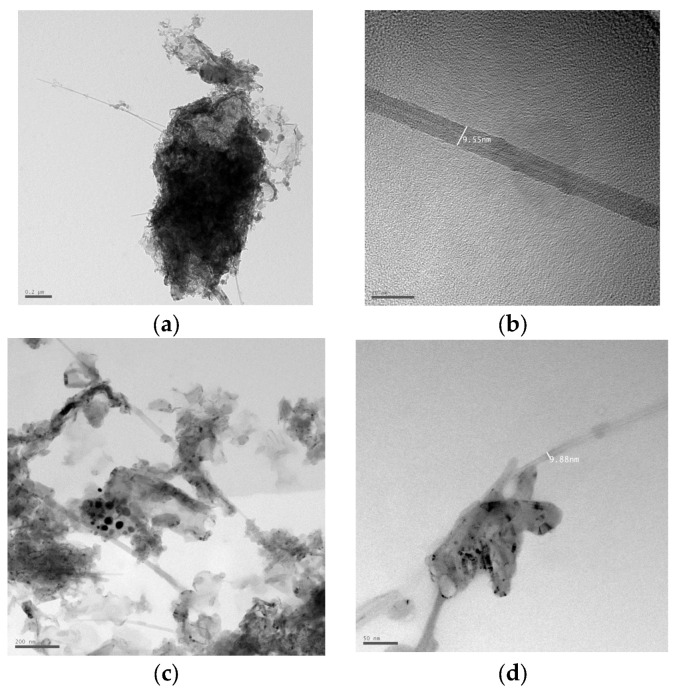
Images of products in the cathode deposit formed in pure ethanol, obtained using HRTEM (**a**) cathode deposit for analysis, total vial (scale 0.2 µm); (**b**) multi-walled nanotube (scale 20 nm; (**c**) general view of the cathode deposit with nano products scale 200 nm; (**d**) multi-walled nanotube (scale 50 nm).

**Figure 12:**
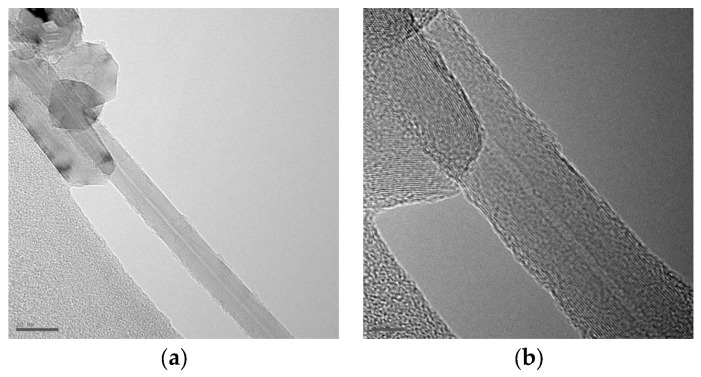
Images of products in the cathode deposit formed in pure propanol, obtained using HRTEM at different magnifications: (**a**) scale bar 20 nm; (**b**) scale bar 5 nm.

**Figure 13:**
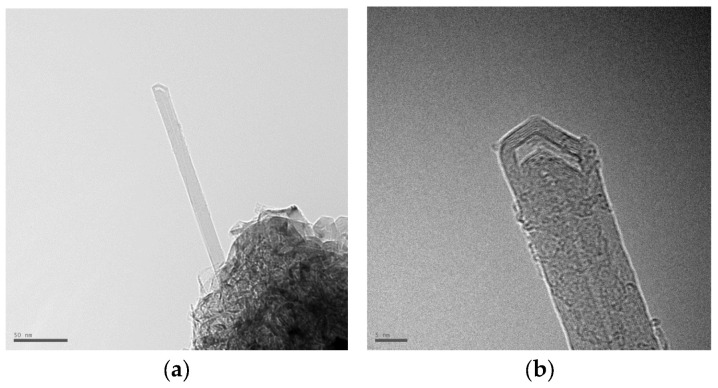
Images of products in the cathode deposit formed in steam plasma (ethanol), obtained using HRTEM (**a**,**b**) multi-walled nanotubes with a lock.

**Figure 14:**
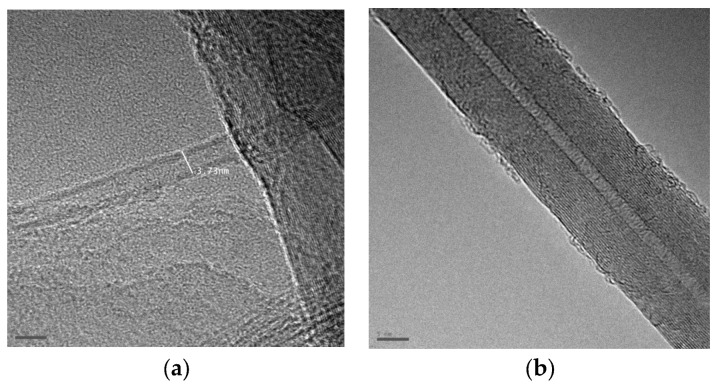
HRTEM images of products in the cathode deposit formed in propanol vapor plasma (**a**) single-wall nanotube (scale 2 nm); (**b**) multi-wall nanotube. (scale 5 nm).

Figure 11 shows the structure of multi-walled carbon nanotubes synthesized by the arc discharge method with various alcohol solutions obtained using HRTEM. Figure 11a,b show a multi-walled nanotube with a diameter of 9.5 nm. Figure 11d shows a multi-walled nanotube with a diameter of 9.88 nm. Concentric-type multi-walled carbon nanotubes (c-MWNTs), in which single-walled carbon nanotubes with a regularly increasing diameter are coaxially arranged (according to the nesting doll principle) in a multi-walled nanotube in Figure 11b,d. The study of various samples showed that most MWNTs have an average diameter of 5–40 nm and a length of 0.1–8 μm. Figure 12 shows a longitudinal projection image of a concentric multi-walled carbon nanotube (c-MWNT) obtained in a propanol medium, obtained using high-resolution transmission electron microscopy. The ends of the multi-walled tubes at different magnifications obtained using HRTEM are shown in Figure 13. HRTEM images of the products in the cathode deposit formed in the vapor plasma of propanol, obtained in Figure 14a single-walled nanotube; Figure 14b multi-walled nanotube.

**Figure 15:**
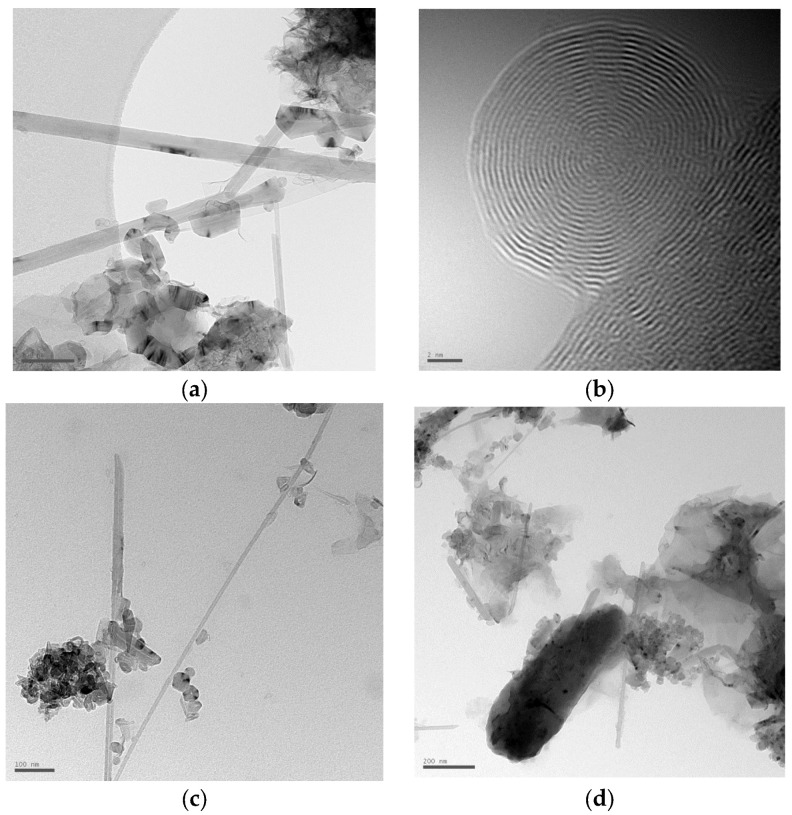
Image of the cathode deposit (plasma composition: 100% benzene), obtained using transmission electron microscopy. HRTEM, (**a**) multi-walled nanotubes with fullerene-like structures (scale 50 nm); (**b**) fullerene on the surface of a multi-walled nanotube (scale bar 2 nm); (**c**) fullerene-like structures on the surface of nanotubes (scale 100 nm); (**d**) cluster of fullerene-like nanostructures (scale 200 nm).

It is known [3,4] that temperatures of 1200 °C or higher are required for the synthesis of fullerenes to convert the carbon source into the gas phase and effectively form molecules. Fullerenes are practically absent in the cathode deposit. Fullerene synthesis occurs in the temperature range of 2500–3500 K [70,71,72,73,74,75,76,77,78]. In our experiments, the temperature is very high, so the synthesis of fullerenes is possible. For example, in [63], plasma temperatures were measured in a solution of propanol, ethanol, and pure water. The arc temperature of water was the highest (6900–7200 K), since the decomposition energy of water molecules to break the O–H bond required a smaller value than for other molecules. The plasma temperature in ethanol was slightly lower (6400–6500 K). On the contrary, two solutions, namely 1-propanol and 2-propanol, showed the lowest arc temperatures among all solutions (5400–5900 K). This is explained by the higher decomposition energy during the rupture of C–H, C–O, and C–C bonds, including the O–H bond. Thus, it was also proven that the arc temperature can be controlled using different types of solutions. The minimum plasma temperature increased with increasing arc current in the range of 5700–8200 K. HRTEM images of the products (nanotubes and fullerenes) in the cathode deposit formed in pure benzene are shown in Figure 15. In particular, fullerenes were identified on the surface of multi-walled nanotubes (Figure 15a–c). The main challenge in fullerene synthesis is extracting fullerenes from the crude carbon black obtained during the synthesis process.

**Figure 16:**
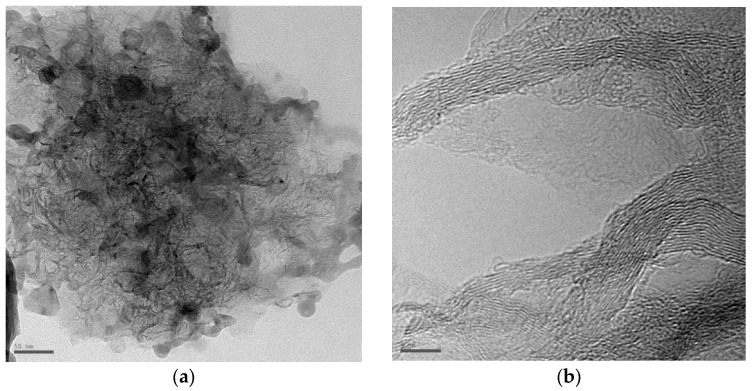
High-resolution transmission electron microscopy (HRTEM) image showing that the deposits are graphene. Scale bars represent 50 nm (**a**) and 5 nm (**b**).

TEM images of the products in the cathode deposit formed in pure benzene are shown in Figure 16. The cathode deposit collected from the cathode included multilayer graphene, carbon onions, and multi-walled carbon nanotubes, in contrast to the deposit on the side surface of the cathodes, which contained only graphene flakes. Figure 16a shows the presence of several types of products: semi-graphite particles, graphene flakes, and spherical particles. The semi-graphite particles have characteristics of graphitized forms of soot [29]. The size of the semi-graphite particles is often in the range of 70–200 nm, and the number of graphite layers can vary from 10 to 100. Graphene flakes are shown in Figure 16a. These flakes have a size (length and width) in the range of 10–100 nm and appear as irregularly twisted flakes that overlap and aggregate, hence they are called “crumpled paper” similar to soot [29]. TEM image analysis shows that the graphene flakes are composed of 1 to 8 graphite layers. The HRTEM image in Figure 16b gives a typical edge image of the graphene flakes, in which the number of graphene layers is 7 and 12, respectively. However, these graphite layers are distorted to some extent, indicating the presence of some disordered structure in the graphene flakes. The spherical particles have a diameter in the range of 20–30 nm (Figure 16a). These particles aggregate and merge together to form a branched morphology.

In [26], a mechanism for the formation of graphene sheets in thermal plasma was proposed. The synthesis products obtained in our work are qualitatively identical to the products described in [26]. For example, spherical particles contain many small and distorted graphite layers, which implies a formation mechanism at low temperatures similar to the typical process of producing soot [58]. Semi-graphitic particles possess graphitized forms of soot [26,58]. The particles are formed via a mechanism of homogeneous (or spontaneously heterogeneous) nucleation of particles with low vapor pressure and then agglomerate/grow via a competing collision–coalescence mechanism between the forming nanoparticles [58]. Such particles undergo a two-step process in thermal plasma [58]: primary particle growth at low temperatures with the formation of spherical particles and graphitization of particles in high-temperature regions, leading to a polyhedral morphology and a shell-like appearance. Graphene flakes are primarily composed of ordered graphite layers, which corresponds to the high formation temperature [26]. The distorted structure in the graphite layers arises due to the formation of five-membered rings [26].

In thermal plasma of plasma torches [67,68,69], there is a non-uniform temperature distribution due to plasma fluctuations. The non-uniform temperature distribution leads to the formation of a low-temperature region (at the periphery) of the plasma column in the nozzle, which leads to the formation of spherical particles. An important clarification in relation to our results regarding the use of benzene is that changes in the enthalpy of the gas can be an important factor influencing changes in the morphology of the product. Thin and straight graphene flakes with the addition of H_2_ [26,27,28,29] play a key role in the synthesis of graphene flakes. It is believed [26] that H atoms can effectively break dangling carbon bonds, forming carbon–hydrogen bonds, and thus prevent the folding and closure of graphite layers. In addition, H atoms perform other functions, for example, etching amorphous carbon [26].

The nanostructured products obtained in our experiments with various alcohol solutions were studied using modern nanostructure identification methods. It was shown that the thermal steam plasma process with various alcohol solutions has great potential for the synthesis of nanotubes and graphene flakes due to its continuous and simple implementation, low-cost raw materials, and adjustable carbon content by combining different mixture compositions. The use of a steam-water plasma torch has virtually no power input limitations, enabling the intensive evaporation of large quantities of carbon and the production of large quantities of starting material (carbon atoms and ions) for subsequent fullerene synthesis. Creating a turbulent zone of sufficient volume with temperatures suitable for fullerene synthesis increases the likelihood of fullerene molecule formation. The presence of some thermal stability in the resulting molecules facilitates their escape from the “hot” synthesis zone. At the same time, a number of fundamental questions remain. One of them is how does an electric arc burn? Initially, electron emission occurs from the tungsten cathode, but it subsequently becomes coated with a carbon film, and the deposit begins to grow. It is likely that the carbon deposit begins to act as a center for thermal emission, maintaining stable arc combustion. This mechanism will be elucidated in future research.

**Figure 17:**
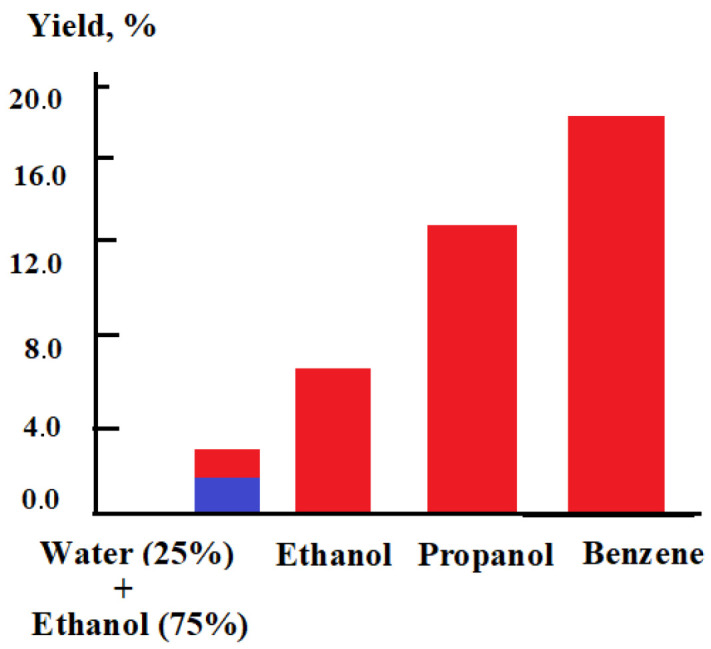
Yield of carbon nanotubes from various monohydric alcohols.

During the research conducted, it was possible to obtain some comparative data on the use of various alcohols (Figure 17 and Table 1). Of course, this is not yet final data and will be adjusted in the future.

At the same time, in our future work, we need to conduct additional research to assess the influence of oxygen and hydrogen in mixed water–alcohol solutions on the formation of nanotubes and graphene flakes. We also need to clarify the yield values for the final product for multi-walled and single-walled nanotubes. Furthermore, it is important to further investigate the effect of water in a mixed water–alcohol solution on nanotube growth. The presence of water in a mixture of alcohol solutions leads to increased catalytic efficiency and the formation of a dense layer of carbon nanotubes. Another parameter requiring separate study is the synthesis rate/yield of the target product, which is an important indicator for evaluating the thermal plasma process. It is necessary to evaluate the synthesis rate/yield of carbon nanomaterials during thermal steam plasma with various alcohol solutions.

In conclusion, we note that in this work, steam-arc plasma is successfully used to obtain carbon nanomaterials by decomposing alcohol solutions. It was demonstrated that the electric arc burns stably in aqueous and alcohol solutions, creating conditions conducive to the synthesis of nanostructured materials. The products obtained in various solutions (namely, ethanol, propanol, and benzene) were characterized using TEM, HRTEM, Raman spectroscopy, and XRD. The experimental results show that the microstructure of the cathode product depends on the composition of the vapor plasma (alcohol solutions).

## 4. Conclusions

1. In an ethanol atmosphere, three types of products coexist in the cathode deposit (semi-graphite particles, multi-walled nanotubes with diameters ranging from 9 to 35 nm, and single-walled nanotubes with diameters of 2–4 nm).
2. In a propanol atmosphere, four product types coexist in the cathode deposit (semi-graphite particles, multi-walled nanotubes with diameters from 10 to 35 nm, single-walled nanotubes with diameters from 2 to 5 nm, and graphene flakes with 1–7 layers).
3. In a benzene atmosphere, five product types coexist (semi-graphite particles, multi-walled nanotubes with diameters from 15 to 45 nm, single-walled nanotubes with diameters from 3 to 5 nm, fullerenes, and graphene flakes with 1–12 layers).
4. Overall, this study demonstrates that the thermal vapor-arc plasma process from the decomposition of alcohol solutions has great potential for the synthesis of nanotubes and graphene flakes, as the morphology and composition of the products can be effectively controlled by varying the plasma composition.

## Figures and Tables

**Table 1:** General parameters of nanostructures after synthesis.

№	Sample (Raw Material)	Type	Synthesis Time, min	HRTEM, SEM (Carbon Nanotubes)	
				D, nm	L, µm;
1	Water (25%) + Ethanol (75%)	Spiderweb (MWNTs)	30	4–8	5–10
2	Ethanol (100%)	Spiderweb (MWNTs)	15–30	4–20	10–40
3	Propanol	Yarn (MWNTs, amorphous carbon, carbon nanoparticles, graphene sheets)	1–3	40–80	0.25–5.5
4	Benzene	Powder (MWNTs, amorphous carbon, carbon nanoparticles, graphene sheets)	0.5–1	20–65	5–25

## Data Availability

The original contributions presented in this study are included in the article/Supplementary Materials. Further inquiries can be directed to the corresponding authors.

## Supplementary Materials

The following supporting information can be downloaded at: https://www.mdpi.com/article/10.3390/nano16070409/s1, Figure S1: Cathode deposit after exposure to ethanol, propanol, and benzene. Figure S2: Cathode deposit after exposure to ethanol, propanol, and benzene. Figure S3. Cathode deposit after work in benzene. Figure S4. Cathode deposit after work in propanol (threads, yarn). Figure S5. Cathode deposit after work in propanol (threads, yarn). Figure S6. Cathode deposit after work in ethanol (threads).
