# Supplementary material for: The Influence of the Composition of a Water–Alcohol Solution on the Synthesis of Nanostructures Using a Steam-Water Electric Arc Plasma Torch
Source: Nanomaterials (Basel). 2026 Mar 28;16(7):409. doi: 10.3390/nano16070409 (PMC13074750; doi:10.3390/nano16070409)
Supplement: Supplementary file 1 [file nanomaterials-16-00409-s001.zip › nanomaterials-4189365-supplementary.pdf]

Cathode deposits formed using an alcohol mixture (ethanol, propanol, benzene) as examined with a scanning electron microscope

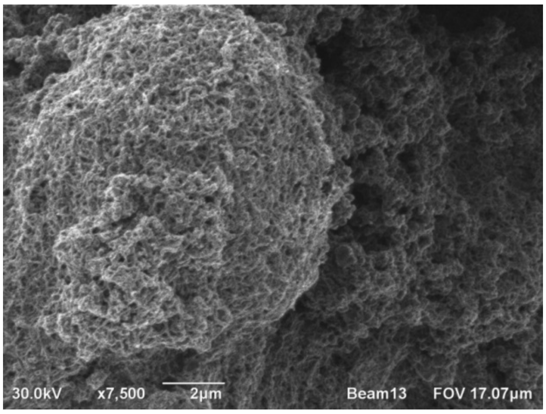

Figure S1: Cathode deposit after exposure to ethanol, propanol, and benzene

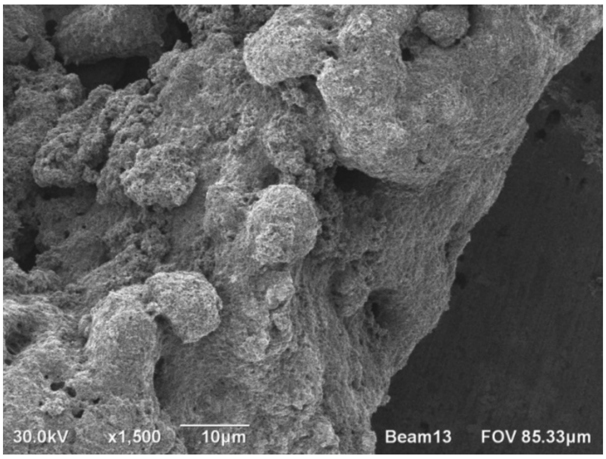

Figure S2: Cathode deposit after exposure to ethanol, propanol, and benzene

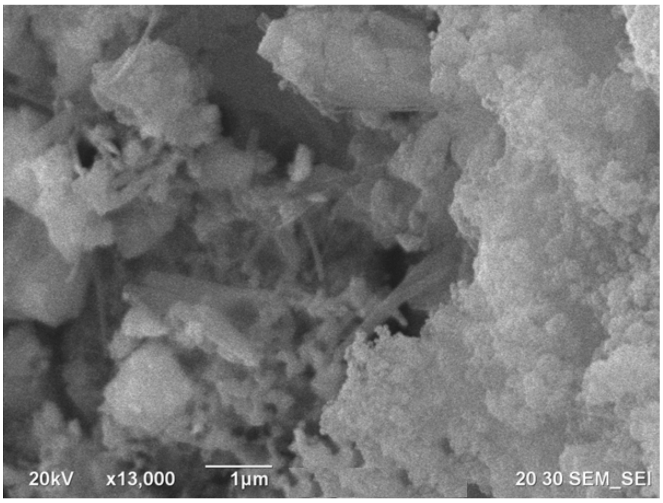

Figure S3. Cathode deposit after work in benzene.

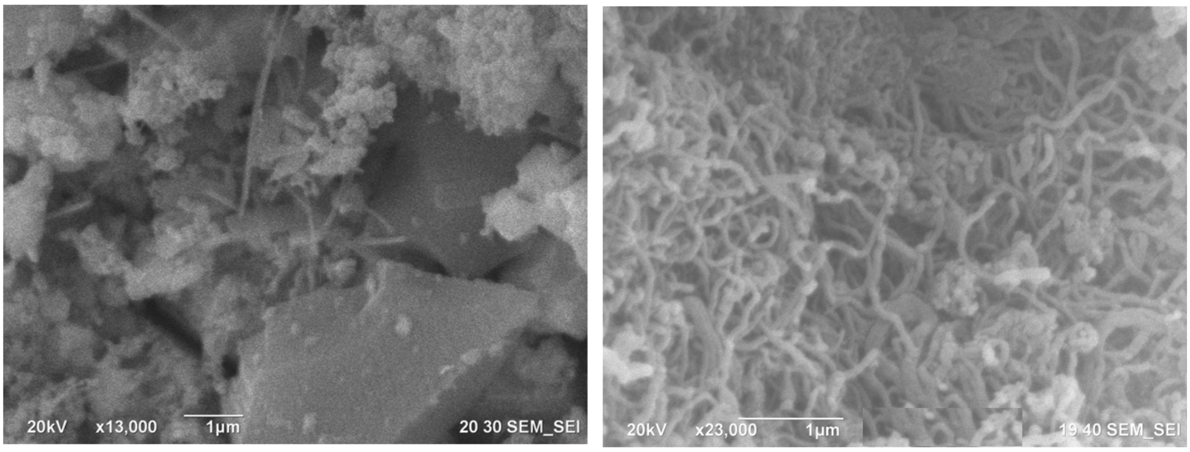

Figure S4. Cathode deposit after work in propanol (threads, yarn).

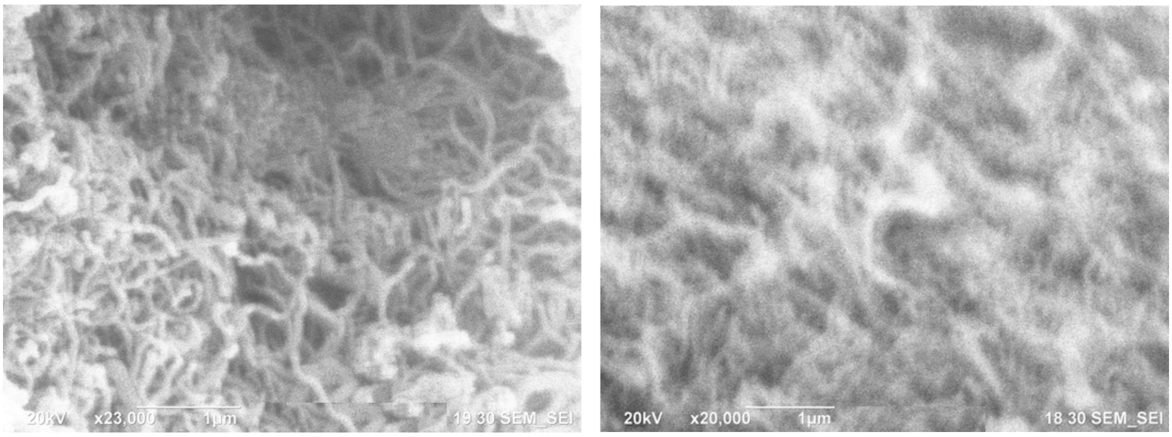

Figure S5. Cathode deposit after work in propanol (threads, yarn).

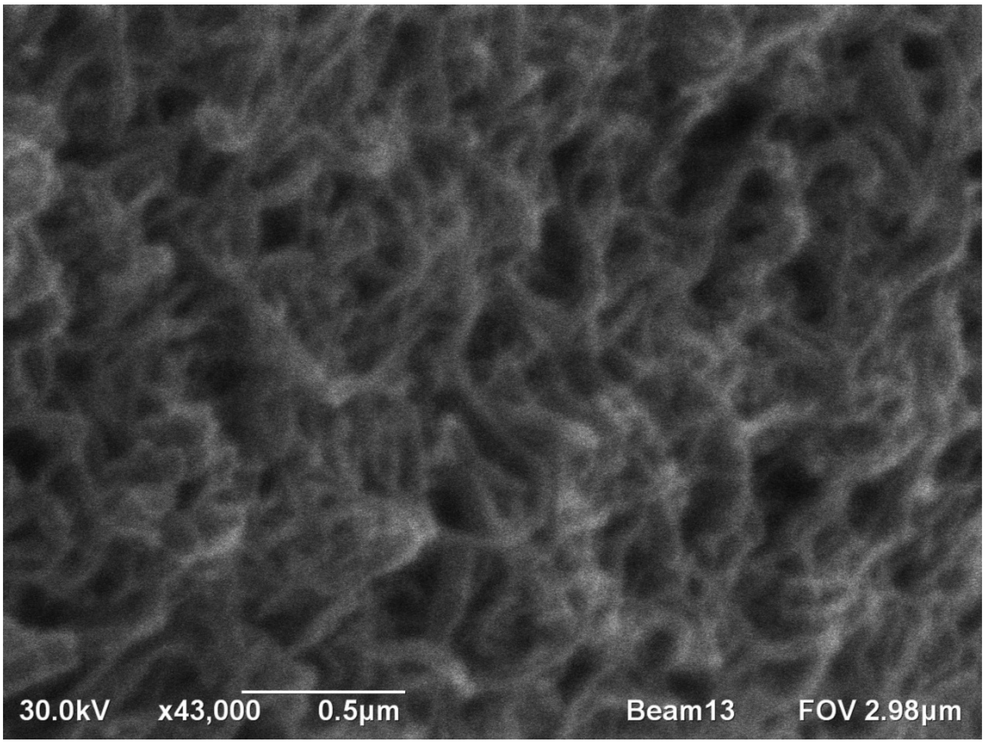

Figure S6. Cathode deposit after work in ethanol (threads).
